# Proceedings of the American Proctological Society

**Published:** 1914-03

**Authors:** Dwight H. Murray

**Affiliations:** Syracuse, N. Y.


### Proceedings of the American Proctological Society.

### Further Observations on Pruritus Ani; Its Probable Etiologic Factor; Results of Treatment.

BY DWIGHT H. MURRAY, M. D., OF SYRACUSE, N. Y.

Dr. Murray's paper, which is a continuation of his investigations on the etiology and treatment of pruritus ani, gave some new points which he had observed during the past year, and his additional experience in the treatment of patients. He found no reason for materially modifying his former reports, but has gathered data which helped to prove the correctness of his previous work. He found streptococcic infection in three cases of pruritus ani and vulvae, and in four cases of pruritus that had involved the scrotum as well as the anus. These complicated cases improved, with the exception of two vulva cases, by the use of the vaccine treatment.

During the past year Dr. Murray has increased his former series of thirty-two cases, by twenty-five additional cases, in five of which streptococcic infection was not found. These cases showed other infections, which still further proves the cocigenous nature of pruritus ani; and also demonstrates that other bacteria than streptococci may bear a casual relationship, as was hinted in his first paper on this subject.

His cases, so far as he has been able to determine, have not been affected by diet. Since Dr. Murray discovered the infection in pruritus ani, he has never interfered with the food of any patient; neither has he restricted them in the smoking or drinking habits. The improvement under the vaccine treatment, without regard to eating, drinking, or smoking, gives him additional proof for the bacterial theory.

During the past year he has carefully investigated as to whether or not the itching extends into the anal canal beyond Hilton's white line, with the result that only in one instance did it extend beyond that point, and then only for a short distance.

His investigations of the past year have given him additional proof that pruritus ani is not caused by any local lesion within the anal canal, and that when such lesions exist with pruritus ani they are coincidental.

In the cases that have been operated on for local lesions, the pruritus ani has not been permanently improved as a result of the operative procedure.

He said that rectal and general surgeons have observed many cases of fistulae with discharges upon the anal skin, without pruritus ani being present. The same is true of hemorrhoids, constipation, and other rectal lesions, pruritus ani occurring in only a small proportion of such cases. He, therefore, still holds that when pruritus ani exists in connection with other lesions that it is a coincidence. In his 1912 report he gave a summary of 900 consecutive rectal cases wherein this fact was established fairly well.

He referred to the opsonic index, or more properly the coefficient of extinction of opsonins, and claimed that much valuable information was to be gained by this test.

His work shows that if a complicating infection exists, and other bacteria than streptococci are found to be the sole invading organisms, we must use the corresponding autogenous vaccine. The opsonic index, following a bacterial diagnosis, is the proper method for determining this.

The results of treatment, and the history of patients, prove to him that if pruritus ani exists with local lesions which demand operation that the prognosis depends upon whether a skin infection is present or not. If the skin infection is present, the local lesions may be cured by the operation, but the patient should not be led to believe that the pruritus ani will also be cured by it. Per contra, if a skin infection does not exist with a local lesion and itching, the prognosis may be that the itching will very likely cease with the cure of the local lesion.

After personal investigation in treating; watching results; noting how cause, effect, and results, dovetail together; comparing these investigations with statements and theories made in text-books, and in articles appearing from time to time in medical journals, and containing no definite pathology or scientific reasons for cause and effect, Murray cannot understand how the profession will uphold such theories, rather than the bacterial theory which has been so well proven in his own cases and confirmed by other observers.

The uniformity of the bacteriologic findings is a strong support for the bacterial theory of the etiology of pruritus ani. The chronicity of all the cases; the uniform symptoms; the similar conditions of the skin; the locality; the regularity as to the time of attacks; the uniformity of itching outside of Hilton's white line; the uniform blood findings as to the coefficient of extinction of opsonins; and the fact that all local applications which have given beneficial results in the past have contained a strong germicide; all point directly to a common cause. Further confirmation is found in the uniformly good results of treatment with autogenous vaccine of the variety of bacteria against which the patient has a low phagocytic power; and in the lack of good results by the various haphazard methods of treatment in general vogue.

His reference to fissures in previous papers having been misunderstood by some, he desired to state that he had referred only to fissure-like cracks of the skin, and not to anal fissures or ulcers.

Endo's medium is used to plate the cultures. The vaccine employed is of the strength of one billion to the c.c., beginning with two minims, or one hundred and thirty millions.

Dr. Murray refers to a paper written by Dr. Jerome Wagner, of New York City, published in the May number of the *Medical Review of Reviews,* in which Dr. Wagner reports some erroneous ideas claimed to have been gleaned from reading Murray's first two reports. Dr. Wagner not having been able to confirm these reports, Dr. Murray pointed out the errors of technique in Dr. Wagner's work, as well as his errors in the interpretation of the reports.

Dr. Murray gave statistics, in favor of his theory, drawn from three years original work on the subject; he also gave a summary of the results of treatment, showing the favorable clinical results with autogenous vaccines in a large majority of the cases treated.

He summed up his conclusions as follows:

1. Results of the past year's work continue to uphold the correctness of the bacterial theory of pruritus ani.

2. It is advisable to make a bacteriologic examination of all cases of pruritus vulvae; also of cases of scrotal pruritus.

3. The coefficient of extinction of opsonins is a valuable aid in diagnosis in complicated and obstinate cases.

4. Pruritus ani in this series of cases rarely extends above the white line of Hilton, and it is still sub judice.

5. The presence of a skin infection with a local lesion begets an unfavorable prognosis for the cure of pruritus ani by an operative procedure.

6. The absence of a demonstrable skin infection and the presence of a local lesion, with pruritus ani, will justify us in making a favorable prognosis for the cure of the pruritus ani by an operative procedure.

7. Pruritus ani, with such infection as we have demonstrated, and a lesion existing in the anus or rectum, according to his statistics, is a coincidence; and the latter lesion is not the cause of the pruritus ani.

8. The sphincter muscle does not allow a leakage of rectal mucus upon the anal skin of one who has pruritus ani, except there is a patulous anus, any more than it does in a normal individual who has no pruritus ani. The moisture of the parts is due to a low-grade inflammation of the infected anal skin.

